# IL-4 gene polymorphisms and their association with nematodes infection in Pakistani population

**DOI:** 10.4314/ahs.v22i2.25

**Published:** 2022-06

**Authors:** Kiran Afshan, Khola Sarfraz, Tooba Kayani, Sabika Firasat

**Affiliations:** Department of Zoology, Faculty of Biological Sciences, Quaid-i-Azam University, Islamabad, 45320, Pakistan

**Keywords:** Interleukin-4, SNPs, Nematodes, Risk Factors, Pakistan

## Abstract

**Background:**

Interleukin-4 (IL-4) plays a central role in the humoral immune defense against nematode parasite infections, inducing IgE switch and regulation of worm expulsion from the intestines. The present study aimed to investigate the polymorphisms in IL-4 gene and their association with socio-demographic and environmental factors among patients with gastrointestinal complaints.

**Method:**

The screened population comprised 305 patients aged 3–50 years from Rawalpindi and Jhelum districts of Pakistan. A well-prepared questionnaire was administered to collect data on socio-demographic and environmental factors. The data were analyzed by using multiple logistic regression models. Molecular analysis was done on 88 confirmed cases passing worms and eggs in stool by using PCR to amplify IL-4 gene.

**Results:**

The result showed higher GI nematodes prevalence in Rawalpindi 34.87% and Jhelum 23.1% among gastrointestinal patients. The multivariate logistic regression model showed significantly (p<0.05) increased risk of infection in participants who were residing in rural areas (OR=321.94; 22.5), having poor economic status (OR=0.34), consuming raw/unwashed vegetables (OR=1.73; 15.39) and did not practice handwashing (OR=2.77; OR=0.30). Sequence analysis showed three novel polymorphisms at SNP g.704_705 ins T, g.3763_3764 ins AC and g.3792 G >A in patients with acute severe infections. Two known polymorphisms SNPs g.8455A>G and g.8492C>A were found in the intron region.

**Conclusion:**

IL-4 gene polymorphisms showed disease susceptibility and consuming raw/unwashed vegetables, poor handwashing practices and poor economic status were the most associated factors with the disease.

## Introduction

Helminth infections display a high ratio of natural variation, with some individuals appearing to be susceptible to the infection 1, whereas others being resistant to infection despite constant exposure.^2^ Host genetic systems play a crucial role in different immunological responses to nematode infection. Involvement of host genetics on disease severity, susceptibility and long term infections are possibly related with variations in immune related gene.^3^ There is considerable evidence for hereditary variation in human populations 4 and farm animals 5 in resistance to nematode infections.

Intestinal nematodes are among the major global disease burden with approximately two billion people infected globally.^6^ Infections by these nematodes alone are estimated to cause the loss of 4.98 million Disability Adjusted Life Years (DALYs) worldwide.^7^ Intestinal nematode infections are widely distributed in tropical and subtropical regions. In Pakistan parasitic infections greatly affect 0.17–0.4 billion middle aged children yearly.^6^ These pathogenic infections are usually not fatal; they are associated with high morbidity rates.^3^ Many individuals develop chronic infection, which leads to anemia, malnourishment and poor cognitive development, resulting in serious health and socio-economic consequences in developing countries.^8^

Polymorphisms in cytokine genes can influence the immune response.^9^ Cytokine genes such as interleukins are natural candidates because of their vital regulatory role in susceptibility to the helminth parasites.^1^ These genes have evolutionary significance as a target of balancing selective processes.^10^ Immunity to enteric parasitic nematode infections is chiefly interceded by T helper cell type2 (CD4) cytokines and TH2 immune response, mainly dependent on cytokine Interleukin-4 (IL-4).^11^,^12^ IL-4 is an important immune-regulatory cytokine which regulate the differentiation of precursor TH cells into humoral mediated T helper cell type subset (TH2).^13^ It initiates the production of immunoglobulin E (IgE) in B-lymphocytes and act as an important regulator of IgG isotype switching, crucial in anti-parasitic immunity.^14^ IL-4 also elevates the synthesis of immunoglobulin IgG and IgA which leads to production and activation of eosinophils which are an important host defense mechanism against many helminths.^15^

Increasing evidence indicates that the human IL-4 gene is polymorphic, variants in this gene have been reported to be associated with altered IgE and IgG levels 16–19 and can affect various functions of cytokine and balance of TH-1 & TH-2 ratio.20 So far numerous variations in IL4 gene have been reported in different diseases i.e. +33C/T (rs2070874), -34C/T (rs2070874), -524C/T, −590C/T (rs2243250), 589 C/T, +3437C/G (rs2227282) and 2979G/T (rs2227284).^9^,^16^,^18^–^20^ Most of them are situated in promoter region and associated with regulation of IgE synthesis.^18^,^21^,^22^ Hence, IL-4 polymorphism can influence the intensity of several infections^18^,^19^,^23^,^24^, including enteric pathogens.^20^,^25^,^26^ Empirical evidence of the importance of IL-4 on the intensity of parasitic nematode infections in human population is still lacking despite its fundamental role in the regulation of parasitic infection and evolutionary importance of IL-4 SNPs.^10^

The present study aimed to find polymorphisms in IL-4 gene among patients infected with gastrointestinal (GI) nematodes from Pakistan. Number of studies have been conducted on functional significance of IL-4 polymorphisms in different diseases worldwide, to our knowledge this is the first study conducted in Pakistan where we investigated the role of IL-4 variants against nematode infection in the local population. The prevalence along with socio-demographic and environmental risk factors was studied to determine the disease association.

## Materials and Methods

### Study Design

A cross-sectional study was conducted in local hospitals of the district, Rawalpindi and headquarters hospital (DHQ) of Jhelum in Punjab province of Pakistan from March 2018 to February 2019. The participants involved in this study belonged to diverse backgrounds.

### Sample size calculation and sampling procedure

The sample size was determined by using the formula: n =Z2P (1-P)/d2 27, where n was the sample size, Z was statistics corresponding to level of confidence, P expected prevalence and d was precision. The sample was calculated by taking the prevalence to be estimated at 50% that gives the maximum sample size, with 95% level of confidence and 5% bound on the error of estimation. Therefore, a minimum sample of 356 gastroenteritis patients was calculated. Using consecutive sampling techniques, 700 individuals were approached with a self-generated questionnaire, and 143 included from Jhelum and 162 patients were from Rawalpindi. Participants of study were aged between 3–50 years. Those included were patients visiting the various outpatient departments (OPDs) with complaints of vague abdominal pain, nausea, vomiting, indigestion, constipation and diarrhoea. Informed written consent was obtained from all the subjects. Those who did not show any signs and symptoms related to parasitic infection, not willing to give 3ml of blood and refused to sign the consent form were excluded.

### Ethical consideration

The ethical approval for this study was obtained from the Bioethical Committee of Quaid-i-Azam University (QAU), Islamabad. Informed written consent was signed by participants and parents consented for children below 18 years of age before blood collection.

### Questionnaire Administration

After completion of the consent process, questionnaires (Supplementary file 1) were administered to the participants and questions were asked in their local language. The information included in questionnaire was: biodata, education level, economic background, family size, and hand washing practices, soil eating habits, sanitation facility, footwear, fingernail status and medical complications. The economic status was assessed by household assets, housing materials, ownership of land or livestock and on monthly household income in local currency. We adapted questions from the Demographic and Health Surveys (DHS) questionnaires (https://dhsprogram.com/). Body mass index was calculated and weight is divided into three categories.

### Blood Sample Collection and Processing

3ml peripheral venous blood was collected from 33 confirmed positive cases passing worms and eggs in stool from Jhelum and 55 confirmed cases from Rawalpindi. Blood was taken in EDTA coated vacutainers and was stored at -20°C for further analysis.

### Molecular analysis

Genomic DNA was extracted by using the Phenol-chloroform organic method described by Sambrook et al.^28^. For primer designing of IL-4 gene, Primer3 input software V.0.4.0 (http://bioinfo.ut.ee/primer3-0.4.0) was used (see table 1). 25 µl of PCR reaction comprises 2.5 µl of sample DNA, containing 0.3 µl of Taq DNA Polymerase recombinant (5 U/*µ*L), 2.5 µl of dNTP Mixture (2.5 mM), 0.5 µl of each primer (10 pmol/µl), 5 µl of 2.5 mL 10x Reaction buffer B (without MgCl2) and 2.5 µl of MgCl2 (25 mM). The PCR thermal cycler was programmed at initial denaturation temperature 96°C for 5 min, denaturation temperature 95°C for 45 Sec (35 cycles), and annealing temperature 52°C–57°C (according to melting temperature of each primer pair) for 1 min, extension temperature 72°C for 1 min and a final extension temperature 72°C for 10 min. The amplified product was run on 2% agarose gel and PCR amplicons were purified by using commercially available kits (WizPrepTM Gel/PCR Purification Mini kit, Seongnam 13209 Korea). The samples were sent for sequencing to DNA Core Facility, Centre for Applied Molecular Biology (CAMB), Lahore Pakistan.

**Table 1:** List of primer pairs used for amplification of genomic region of selected exons of *IL-4* gene

Gene	Exons	Primer Sequence	Base pairs	Melting Temperature	Product Size
***IL-4***	Exon2-F	GTTGGAACTGGTGGTTGGT	19	58.23	488bp
	Exon2-R	GAGGGCTTTTCTTTGACCAG	20	58.91	
	Exon3-F	TAATTTCCAGGCTCCAAGC	19	57.9	334bp
	Exon3-R	GCAGAGCGGTTAGAAAGACAT	21	58.62	
	Exon4-F	GAGAGGTTGTTGACAGAGGT	20	54.1	559bp
	Exon4-R	TTTAGTGACACGTCCTCAGC	20	56.5	
	Exon5-F	GCTGGCCTAGTGATAGAGAC	20	54.2	518bp
	Exon5-R	TATGTCCCTAACTCGAGAGG	20	54.5	

### Sequence analysis

Sequencing results were interpreted using bioinformatics tools Sequencher v 5.4.6 software (http://www.genecodes.com/content/sequencher-546-released-0) and Chromas v 2.6.5. Reference gene sequence was obtained from Ensembl Genome Browser (https://asia.ensembl.org/index.html). Sequenced data was aligned with reference sequence in CLC Genomics Workbench (v3.6.5) (https://www.qiagenbioinformatics.com/products/clc-genomics-workbench/) and BioEdit (v7.0.5). In case of any conflict in data, bioinformatics tool Mutation Taster (http://mutationtaster.org/) was used for confirmation of the variants.

### Data Management and Statistical Analysis

During collection of data, questionnaires were checked regularly to avoid any logical mistakes or missing information. The data was entered and managed carefully in Microsoft Excel (2007) file. The analysis of risk factors was conducted by multivariate analysis using multiple logistic regression to examine socio-demographic and environmental variables, and determined those that best predict the outcome. Level of significance was set at p≤0.05 or p≤0.01. The data was analysed in SPSS ver.20.

## Results

### Socio-Demographic Characteristics

The study participants belonged to district Rawalpindi, of these 55 (34.0%) were confirmed positive cases and 107 (66.0%) were negative, while individuals from Jhelum 33(23.1%) were confirmed positive and 110 (76.9%) were negative for GI nematode infections. Large number of study participants belonged to rural areas and were divided into four age groups (Table 2).

**Table 2:** Scio-demographic characteristics and prevalence of GI nematodes in the study participants

Characteristics	Rawalpindi		Jhelum	

	Total	Positive	Total	Positive
	n (%)	n (%)	n (%)	n (%)
**Gender**				
Male	69(42.6)	25(15.4)	62 (38.3)	13 (8.0)
Female	93(57.4)	30(18.5)	81 (50.0)	20 (12.3)
**Age**				
3 to 14	123(75.9)	43(26.5)	101 (62)	23(14.1)
15 to 26	19(11.7)	6(3.7)	31 (19)	3 (1.8)
27 to 38	10(6.2)	1(0.6)	8 (4.9)	5 (3.1)
39 to 50	10(6.2)	5(3.1)	3 (1.8)	2 (1.2)
**Weight**				
Normal	59(36.4)	15(9.3)	61 (37.4)	18 (11)
Overweight	11(6.8)	2(1.2)	3 (1.8)	1 (0.6)
Underweight	92(56.8)	38(23.5)	79 (48.5)	14 (8.6)
**Residence**				
Rural	160(98.8)	53(32.7)	96 (67.1)	31 (21.7)
Urban	2(1.2)	2(1.2)	47 (32.9)	2 (1.4)
**Family size**				
3 to 7	125(77.2)	44(27.2)	127(88.8)	33(23.1)
>7	37(22.8)	11(6.8)	16 (11.2)	0 (0)
**Economic status**				
Middle	61(37.7)	20(12.3)	76 (53.1)	16(11.2)
Poor	101(62.3)	35(21.6)	67 (46.9)	17(11.9)
**Education of** **Subject**				
Uneducated	7(4.3)	4(2.5)	24 (16.8)	2 (1.4)
Pre-School	29(17.9)	14(8.6)	4 (2.8)	2 (1.4)
Primary	79(48.8)	25(15.4)	67 (46.9)	18(12.6)
Secondary	40(24.7)	10(6.2)	25 (17.5)	4 (2.8)
Tertiary	7(4.3)	2(1.2)	23 (16.1)	7 (4.9)

### Multivariate Conditional Logistic Regression Analysis of Case and Control groups

The multiple conditional logistic regression model in Rawalpindi District shown in table 3. The infection rate was significantly increasing in rural areas [OR= 321.949, 95% CI: 9.203-111262.67, p= 0.001] as compared to urban areas. The socio-economic status of individuals was found to be a significant risk factor as the infection rate was decreasing in participants having average socio-economic status [OR=. 0.342, 95% CI: 0.118-0.992, p= 0.048]. Contact with animals showed non-significant association with nematode infection but still more infection rate [OR= 2.57, 95% CI: 0.916-7.221, p= 0.073] was found among patients who had contact with animals as compared to those patients who had no contact with animals. The multiple logistic regression analysis indicated type of houses, exposure to animal and human excreta and presence of animals as non-significant (p>0.05) risk factors for nematode infection. Two-time higher risk of infection [OR= 2.838, 95% CI: 0.943-8.543, p= 0.063] was found among patients who were uneducated as compared to educated participants. Source of drinking water factor was significantly associated with nematode infection as infection rate was three-time increasing among those patients who had hand pump water as a source of drinking water [OR= 3.316, 95% CI: 1.586-6.932, p= 0.001]. Hand washing practice after toilet use was significantly associated with nematode infection [OR= 2.775, 95% CI: 1.124-6.520, p= 0.027]. Significant association was found between infection and hand washing before eating [OR= 0.301, 95% CI: 0.145-2.096, p= 0.001]. Hand washing after work was found to be a non-significant factor [OR= 0.868, 95% CI: 0.359-2.096, p= 0.754. Significant risk decrease was observed in participants who had trimmed nails compared to the ones who did not trim nails regularly [OR=0.196, 95% CI: 0.037-1.03, p= 0.05]. A significant increase in infection [OR=1.731, 95%CI: 0.06-44.20, p=0.01] was found in participants consuming raw unwashed vegetables as compared to those consuming washed vegetables. Infection rate was increasing among individuals consuming leftover food [OR= 9.347, p= 1.00]. The result showed the habit of nail biting, finger nail hygiene and thumb sucking was not significantly (p>0.05) associated risk factors.

**Table 3:** Multivariate logistic regression model to analyze GI nematode association of socio-demographic and environmental risk factors among case and control groups

Variables	Rawalpindi					Jhelum				
	
	GI Nematodes Prevalence		Crude OR	Adjusted OR	p value	GI Nematodes Prevalence		Crude OR	Adjusted OR	p value
	Case	Control	(95% CI)	(95% CI)		Case	Control	(95% CI)	(95% CI)	
**Weight**										
Normal	17	22	Reference			18	13	Reference	Reference	
Underweight	38	33	1.87(0.87–3.99)	0.93(0.32–2.67)	0.901^NS^	15	20	0.54 (0.20–1)	0.43 (0.095–2)	0.28^NS^
**Residence**										
Urban	2	28	Reference			2	17	Reference	Reference	
Rural	53	27	46.41(5.91–364.10)	321.94(9.20–111.26)	0.001**	31	16	24.35(3.0–197)	22.5 (2.04–271)	0.011*
**Economic** **status**										
Middle	20	28	Reference			17	20	Reference	Reference	
Poor	35	27	0.57(0.27–1.20)	0.34(0.11–0.99)	0.048*	16	13	0.59(0.18–2)	0.16 (0.005–5)	0.29^NS^
**Type of houses**										
Concrete	27	29	Reference			18	17	Reference	Reference	
Semi-concrete	25	10	0.72(0.44–1.17)	1.45(0.45–4.62)	0.523^NS^	9	6	0.80(0.44–1)	0.54 (0.0138–21)	0.74^NS^
Non-concrete	3	16				6	10			
**Contact with** **animals**										
No	22	30	Reference			18	22	Reference	Reference	
Yes	33	25	1.98(0.7–4.53)	2.57(0.91–7.22)	0.073^NS^	15	11	1.77(0.60–5)	2.18 (0.497–96)	0.68^NS^
**Presence of** **Animals**										
Small ruminant	3	12	1.73(1.20–2.49)	2.02(0.77–5.27)	0.148^NS^	11	8	1.44(0.66–3)	0.30 (0.014–6)	0.44^NS^
Large ruminant	20	11				4	3			
Small & large ruminant	9	2								
No	23	30	Reference			18	22	Reference	Reference	
**Source of drinking** **water**										
Public pipeline	1	0	Reference			4	0	1.24(0.75–2)	1 (0.909–5)	0.08^NS^
Boring	16	33	1.44(0.94–2.20)	3.31(1.58–6.93)	0.001**	17	18	Reference	Reference	
Hand pump	22	19				10	12			
Well	16	3				2	3			
**Education** **level**										
Tertiary	2	41	Reference			2	4	0.48 (0.20–1)	0.63 (0.032–13)	0.76^NS^
Secondary	10	7	0.94(0.64–1.37)	2.83(0.94–8.54)	0.063^NS^	2	4	Reference	Reference	
Primary	25	20				18	17			
Pre-school	14	2				4	0			
Uneducated	41	12				7	8			
**Hand washing** **after toilet**										
Water and soap	25	31	Reference			22	18	Reference	Reference	
With only water	17	24	2.14(1.18–3.86)	2.77(1.12–6.52)	0.027**	11	15	0.60(0.22–2)	0.19(0.0244–1.5)	0.11^NS^
No	13	0								
**Hand washing** **before eating**										
Water and soap	25	6	Reference			9	9	Reference	Reference	
With only water	17	8	0.23(0.123–0.431)	0.30(0.14–2.09)	0.001**	2	3	1.05(0.54–2)	1.98(0.636–6)	0.23^NS^
No	13	41				22	21			
**Hand washing** **after work**										
Water and soap	18	4	Reference			8	3	Reference	Reference	
With only water	6	9	0.32(0.16–0.65)	0.86(0.35–2.09)	0.754^NS^	6	6	0.4(0.17–1.11)	0.098(0.145–0.7)	0.018*
No	31	42				19	24			
**Finger nail** **status**										
Short	44	28	Reference			20	22	Reference	Reference	
Medium	11	27	0.271(0.115–0.637)	0.19(0.03–1.03)	0.054^NS^	13	11	1.33(0.46–4)	2.42(0.479–12)	0.28^NS^
**Habit of nail** **biting**										
Yes	11	8	1.45(0.541–3.93)	1.09(0.23–5.10)	0.912^NS^	9	7	1.38(0.45–4)	1.90(0.435–8)	0.39^NS^
No	44	47	Reference			24	26	Reference	Reference	
**Eating** **raw/unwashed** **vegetables**										
Yes	17	30	0.321(0.135–0.76)	1.73(0.06–44.20)	0.01**	20	13	2.87(0.94–9)	15.39(1.516–156)	0.021*
No	38	25	Reference			13	20	Reference	Reference	
**Finger nail** **hygiene**										
Clean	33	29	Reference	3.79(0.77–18.56)	0.10^NS^	20	21	Reference	Reference	
Dirty	22	26	0.73(0.33–1.59)			13	12	1.20(0.366–4)	0.157(0.0141–2)	0.13^NS^
**Eating leftover** **food from day** **before**										
Yes	51	53	0.48(0.86–2.73)	9.34(0)	1.00^NS^	32	31	2(0.181–22)	2.42(0.121–49)	0.56^NS^
No	4	2	Reference			1	2	Reference	Reference	

Multivariate logistic regression analysis for risk factors associated with GI nematode infections of Jhelum District are given in table 3. Decreasing risk was found in female patients [OR= 0.27, 95%CI= 0.029-2.611, p= 0.262] when compared to males, but did not differ significantly. The infection was significantly increasing in patients of rural areas [OR= 22.5, 95% CI =2.0479-271.396, p= 0.011] as compared to urban areas. Socioeconomic status [OR= 0.16, 95% CI= 0.005-5, p= 0.298] and literacy rate [OR= 0.63, 95%CI= 0.032-13, p= 0.767] showed no significant association. A decreasing risk of infections was found in individuals living in non-concrete houses [OR= 0.54, 95%CI= 0.0138-21.303, p= 0.744] as compared to individuals living in concrete houses. Risk increase was found in individuals who had contact with animals [OR= 2.18, 95%CI= 0.497-95.793, p= 0.686] as compared to individuals with no contact with animals. Odds ratio showed [OR= 0.30, 95%CI= 0.014-6, p= 0.441] decreased risk of infections in individuals who did not have any animals in their houses. Multivariate conditional logistic regression model showed types of toilets [OR= 0.54, 95%CI= 0.0005-515, p= 0.861] and source of drinking water [OR= 1, 95%CI= 0.909-5, p= 0.084] as non-significant risk factor for GI nematode infection. Odds ratio showed [OR= 0.19, 95% CI =0.0244-1.5013, p= 0.116] that risk decrease was found in individuals who washed their hands with water and soap after using the toilet. Odds ratio calculated for hand washing before eating showed [OR= 1.98, 95% CI=0.636-6.166, p= 0.238] that risk increase was found in participants with no hand washing practices before eating. However, the associations were not significant (p>0.05). Odds ratio [OR= 0.098, 95% CI = 0.145-0.672, p= 0.018] showed that increase in hand washing practice caused a decrease in rate of infections, which is statistically significant. Odds ratio showed [OR= 0.55, 95% CI=0.136-2.236 p=0.405] decreasing risk of infections in individuals with no soil eating habit. Similarly the decreased risk of infections in individuals who had no contact with soil [OR= 0.70, 95% CI= 0.145-3, p= 0.668]. Significantly increased risk was found in individuals eating unwashed vegetables[OR= 15.39, 95%CI= 1.516-156, p= 0.021], when compared to the ones consuming washed vegetables. Odds ratio showed [OR= 2.42, 95% CI= 0.121-49, p= 0.562] that increase in consumption of leftover food caused an increase in rate of infection. The result showed non-significant association between GI nematode infections and risk factors including: shoe wearing habit [OR=1.106, 95%CI=0.312-3.913, p=0.876], finger nail status [OR = 2.42, 95% CI=0.479-12.268, p= 0.284], finger nail hygiene [OR= 0.157, 95% CI=0.0141-1.741, p=0.132] and habit of nail biting [OR= 1.90, 95% CI= 0.435-8, p= 0.392].

### Polymorphism in IL-4 gene Intronic Region

**Figure 1:**
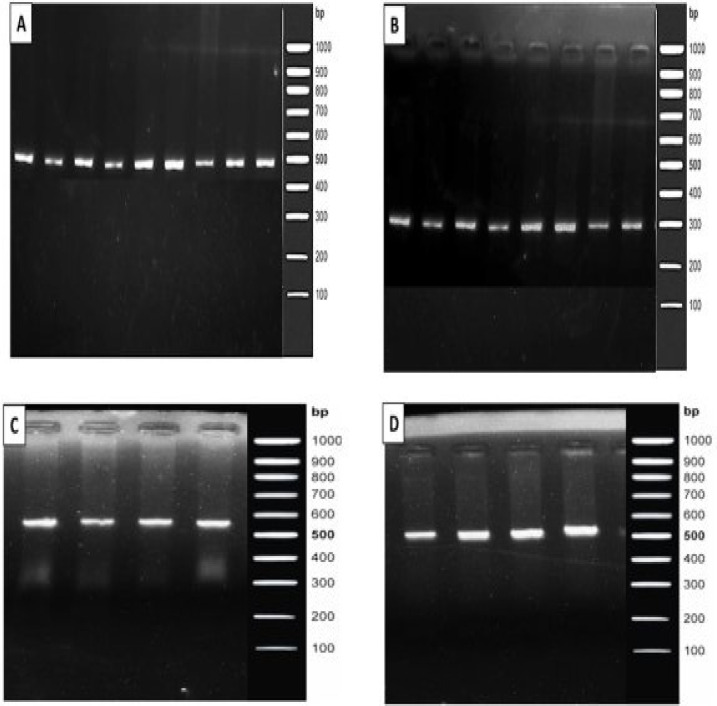
Gel image showing amplified PCR products of flanking regions and exons **a)** 2 **b)** 3 **c)** 4 **d)** 5 of *IL-4* gene in confirmed positive patients.

**Figure 2:**
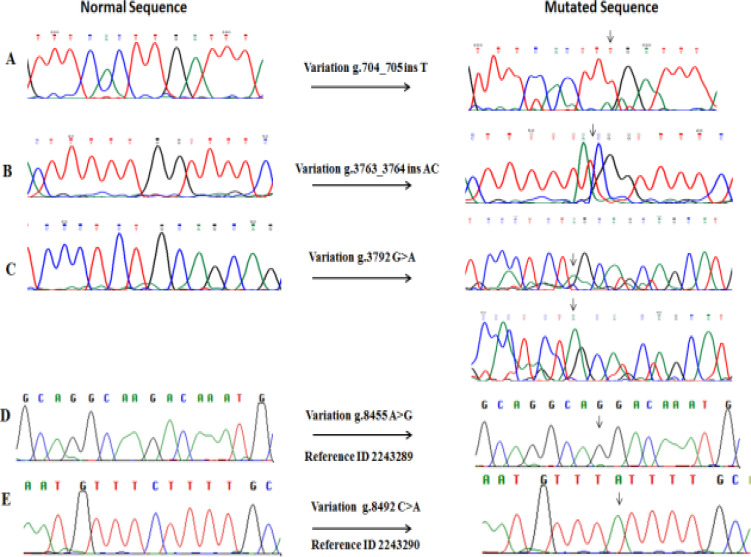
Sequence chromatograms for three novel single nucleotide polymorphisms [**A)** chr5:132010381 _132010382 ins T **B)** chr5:132013440_132013441 ins AC **C**) chr5:132013469 G>A ] and two known polymorphisms [ **D**) ID rs2243289 v. g.8455A>G **E**) ID rs2243290 v.g. g.8492 C>A] were identified in this study. The normal sequence is shown on the left and mutated sequence on the right.

Four selected exons and flanking regions of the IL-4 gene in 88 enrolled patients were sequenced and revealed 8 sequence variations of which 6.8% (6/88) were single nucleotide variations. Amplified PCR product of exon 2, 3, 4, 5 and its flanking region is given in Fig.1. Polymorphism in the flanking non-coding region of IL-4 gene in patients with severe and chronic cases of nematode infection is given in table 4. A novel polymorphism in exon 2 flanking region g.704_705 ins T was detected in one patient (NM2-22) in the intronic region at physical location (chr5:132010381_132010382insT) (Fig. 2a). This polymorphism was present in homozygous condition and did not depict any change in amino acid. According to Insilco Mutation taster analysis this variant might affect protein features through modification of splicing.

**Table 4:** Polymorphism in exon 2, 3,4, 5 and flanking non-coding region of *IL-4* gene in patients with severe and chronic cases of nematode infection (n=88)

Patient ID	Gender	Age (years)	Symptoms	Nucleotide change	Intron/Exon	In Silico prediction	Ref.ID	Novelty
NM2-22	Male	14	Acute/Severe	g.704_705 insT	Intron	Polymorphism	-	Novel
NM3-12	Female	10	Acute/Severe	g.3792G>A	Intron	Polymorphism	-	Novel
NM3-14	Female	28	Acute/Severe	g.3792G>A	Intron	Polymorphism	-	Novel
NM3-31	Female	44	Acute/Severe	g.3792G>A	Intron	Polymorphism	-	Novel
NM3-17	Female	8	Chronic	g.3763_3764 ins AC	Intron	Polymorphism	-	Novel
NM5-1	Female	30	Acute/severe	g.8455(A>G)	Intron	Polymorphism	rs2243289	Known
NM5-9	Male	9	Chronic	g.8455(A>G)	Intron	Polymorphism	rs2243289	Known
NM5-9	Male	9	Chronic	g.8492(C>A)	Intron	Polymorphism	rs2243290	Known

The exon 3 flanking regions showed variation g.3763_3764 ins AC in one patient NM3-17 in the intronic region (Fig. 2b) at physical location (chr5:132013440_132013441insAC). Other polymorphisms g.3792G>A were detected in intron (Fig. 2c) at physical location (chr5:132013469G>A). It was present in heterozygous condition in three patients (NM3-12, NM3-14 & NM3-31). Both polymorphisms might affect protein features possibly through modification of splicing according to mutation taster analysis.

The sequence variations in the flanking (intron) region of exon 5 revealed two variants. One variant was at position g.8455 (A>G) where A base pair is substituted by G in two patients (NM5-1 & NM5-9) (Fig. 2d). This is previously reported SNP with Reference ID rs2243289 and did not have any effect on splice site. The second variant was at position g.8492(C>A), where C base pair was substituted by A in the flanking region in one patient (Fig. 2e). It is previously reported SNP with reference ID rs2243290. Mutation taster predicted this variation might affect the splice site of mRNA of exon 5 and might affect protein structure.

## Discussion

In this study, prevalence of GI nematode infections among patients with gastrointestinal complaints attending local hospitals of Rawalpindi and Jhelum Districts was 34.87% and 23.1% respectively. A first comprehensive nationwide survey was conducted in the country in late 2016, the finding of the survey indicated that Northern regions of Punjab have the highest prevalence, with 56% in Rawalpindi and 31% in Gujarat, respectively, followed by 37% in district of Swat. In contrast, low prevalence was recorded in the southern region of Pakistan, with exception of Karachi where prevalence reached 20%. The most important finding in the survey was that basic hygiene practices and sanitation infrastructure were poor in all study areas.^29^ The present result showed higher prevalence in female (18.5%, 12.3%) as compared to males (15.4%, 8.0%) in Rawalpindi and Jhelum respectively, consistent with other study.^30^ This may indicates that gender play significant role with GI nematode prevalence and explained by the fact that due to lifestyle of particular area, females are more likely to get infected because of their interaction with contaminated food, water and environment.^31^

The current result showed higher infections in age groups of 3–14 years and lowest among the old age groups, in accordance with other studies where prevalence of GI nematode infections is higher in children and young individuals.^32^,^33^ This finding could be attributed to the fact that individuals of this age spend most of their time outdoor, playing with soil, eating unclean food.^33^ Lack of hygiene practices such as lack of hand washing and shoe wearing habit can also be the main determinants of infections at his age.^34^

Underweight and normal individuals have relatively high rate of infection (23.5%, 8.6%) and (9.3%, 11%) respectively and findings showed similarity to previous work from Sri Lanka and India.^32^,^35^,^36^ Insufficient supply of food to individuals, poverty can be reason for high prevalence in underweight individuals.^37^ Prevalence of infections was significantly higher among individuals with family size 3–7 members (27.2% and 23.1%) and not accordance with previous report.^33^ It is reported previously that over crowdedness and large number of family members are associated with high prevalence of parasitic infections.^38^

Rural community (32.7% and 21.7%) showed significantly increased risk of infection and results are consistent with previous study.^39^ Poverty, poor sanitation facilities, open defecation system and low rate of literacy are possible reasons for high prevalence of nematode infection in rural areas.^40^ High prevalence of infections was found in individuals with poor economic status (21.6% and 11.9%) but difference is not significant. These results are in agreement with previous report.^41^ Poor living conditions, lack of sanitation, use of unsafe water supply and improper waste of disposal can be reasons associated with high prevalence of infections in individuals with poor socioeconomic status.^40^ Education of subjects did not show significant association with nematodes, but individuals with low literacy rate showed higher infection, consistent with previous report.^42^ Lack of education about sanitation and hygiene are possible reasons associated with high prevalence of infections.^43^

One of main determinants of intestinal infections is the inadequate disposing of excreta.^44^ Houses without safe disposal of excreta have higher prevalence of infection. Poor hygiene practices and lack of hand washing practices after using the toilet could be reasons for high prevalence of infections in these individuals. The results showed poor personal hygiene plays a major role in transmission of infections. Individuals with no hand washing practices before eating and after work/play showed higher risk of infections compared to those who washed their hands with soap and water. Current findings are similar with previous reports on lack of hand washing practice, no shoe wearing habit, drinking contaminated water, eating of raw/unwashed vegetables/fruits can lead to ingestion of parasitic eggs. 45,46

In the current study, screening of exon 2, 3, 4, 5 and flanking regions of IL-4 gene was done to find the polymorphisms which might be linked with susceptibility to nematode infections. It has been observed in our study that all these patients were suffering from severe infection and suggested that the IL-4 might play a regulatory role in the pathogenesis of nematode infections in these patients. The exon 2, 3, 4 and 5 did not reveal any polymorphisms. The polymorphisms were only recorded in promotor and flanking regions of exons. Three novel polyorphisms were detected, which were not identical to known and functionally relevant IL-4 polymorphisms. The SNP g.704_705 insT was detected in the flanking region of exon 2. This mutation was reported in one patient who had acute severe nematode infection. We also reported two variations in exon 3 and its flanking region, g.3763_3764 ins AC polymorphism was detected in one patient having chronic severe infection. g.3792 G >A polymorphism was detected in three patients with acute severe infections. The current study needs further screening of the number of cases and controls to conduct association studies.

The exon 4 did not reveal any polymorphism. Previously various studies reported SNPs in interleukin 4's promoter region and not in exon 4. These SNPs in IL- 4 promoter region affect its transcription, thus producing altered IL-4 protein levels leading to either higher or lower IgE titres.^16^,^17^ So SNPs in IL-4 gene can impact the intensity of various infections^16^,^18^,^19^,^24^,^47^, including enteric pathogens.^11^,^25^,^48^ Previously complete screening of IL-4 gene was done to study polymorphisms relating to various diseases like asthma and IgE in children but no SNPs were found in exon 4 which could be relating to diseases.^23^,^47^

However previous studies reported SNPs in the flanking (intron) region before exon 4 related to various diseases. In one study, SNPs reported in flanking region before exon 4 were associated with reduced risk of severe tuberculosis.^49^ In another study SNPs in flanking region showed association with asthma.^50^

In present study, two SNPs have been found in the flanking (intron) region before exon 5 in two patients. One SNP was at position 8455 on IL-4 gene, where A base pair is substituted by G. Previously this SNP g.8455A>G with Reference ID rs2243289 was found in a study conducted to find any interaction between IL-4 gene and type 1 diabetes. No evidence of association of this SNP was found with type 1 diabetes in study conducted in European populations.^51^ This SNP is previously reported in study conducted in Thailand to find any polymorphisms in 5q31 gene cluster which might be causing susceptibility to severe malaria but no association was found between this SNP and malaria susceptibility.^52^ This SNP was found in study conducted to compare association of candidate genes with asthma between children of European and African ancestry but no association was found between this SNP and asthma in both populations.^50^

The SNP g.8455A>G is also reported in study conducted to find polymorphisms which are associated with providing resistance to T. cruzi in Bolivian population but no association of this SNP was found with resistance.^53^ The SNP is reported in previous studies conducted in China to find polymorphisms which might be associated with non-infectious disease like renal cell carcnoma and Grave's disease in children but no association of this SNP was found with these diseases.^54^,^55^ The SNP was found in study conducted to investigate association between IL-4 and IL-10 polymorphisms with risk of antituberculosis drug induced hepatotoxicity in Chinese population but no association was found.^56^ However, the SNP g.8455A>G rs2243289 of the IL-4 gene was associated with decreased risk of developing steroid-induced osteonecrosis of femoral head in Chinese population.^57^

In current study, this SNP g.8455A>G was found in two confirmed positive patients of GI nematode infection who had severe signs and symptoms of disease, but association of this SNP in causing susceptibility to nematode infections cannot be done yet as more comprehensive case control study with large number of case and control samples should be conducted to confirm its association with nematode infections.

Second SNP was at position g.8492 on IL-4 gene, where C base pair was substituted by A in the intron region in one patient only. This SNP is reported previously with reference ID rs2243290 in studies conducted to find polymorphisms in IL-4 gene which could be associated with various diseases like trypanosomiasis, cancer, asthma and allergy. This SNP g.8492C>A was reported previously in study conducted in African, American population to study polymorphisms associated with asthma and allergy, but no association was found.^58^ No association of this SNP was found in causing resistance to T. cruzi in Bolivian population.^54^ No significant relationship was found between this SNP and rhinoconjunctivitis in study conducted to find polymorphisms which could be associated with risk of rhinoconjunctivitis in Japanese women.^59^

The SNP g.8492C>A SNP was reported previously in study conducted to find polymorphisms in interleukin genes and association with rectal and colon cancer but no association of this SNP was found with cancer.^60^ One study that was conducted to investigate the polymorphisms in immunity modulating genes which might be associated with invasive Aspergillosis reported the presence of this SNP g.8492C>A in IL-4 gene in patients but no association of this SNP was found with disease.^61^ Various other studies reported the presence of this SNP in IL-4 gene but no significant association of SNP was found in those studies.^62^,^63^ In current study, SNP g.8492C>A was found in single patient of nematode infection only who had severe sign and symptoms of infection. In order to confirm association of SNP g.8492C>A with nematode infection, a more extensive study with increased number of cases and controlled samples is recommended for confirmation.

## Conclusion

The study concluded IL-4 gene polymorphism is linked with disease susceptibility as it may have the ability to cause significant changes in function by altering the levels or activity of specific proteins that balance TH-1 & TH-2 ratio. The current study recommended the need for a large-scale screen of IL-4 gene to find association with GI nematodes. Furthermore, integrated control programs including periodic deworming, improving sanitation, appropriate health education and environmental measures are required to reduce the transmission of disease.

## References

[1] Hotez PJ, Brindley PJ, Bethony JM, King CH, Pearce EJ, Jacobson J (2008). Helminth infections: the great neglected tropical diseases. J Clin Invest.

[2] McSorley HJ, Maizels RM (2012). Helminth infections and host immune regulation. ClinMicrobiol Rev.

[3] Flores J, Okhuysen PC (2009). Genetics of susceptibility to infection with enteric pathogens. CurrOpin Infect Dis.

[4] Quinnell RJ (2003). Genetics of susceptibility to human helminth infection. Inter J Parasitol.

[5] Dominik S (2005). Quantitative trait loci for internal nematode resistance in sheep: a review. Genet Sel Evol.

[6] World Health Organization Soil-transmitted helminth infections.

[7] Murray CJ, Vos T, Lozano R, AlMazroa MA, Memish ZA (2010). Disability-adjusted life years (DALYs) for 291 diseases and injuries in 21 regions, 1990–2010: a systematic analysis for the Global Burden of Disease Study.

[8] Waite RC, Velleman Y, Woods G, Chitty A, Freeman MC (2015). Integration of water, sanitation and hygiene for the control of neglected tropical diseases: a review of progress and the way forward. Int Health.

[9] Micheal S, Minhas K, Ishaque M, Ahmed F, Ahmed A (2013). IL4 gene polymorphisms and their association with atopic asthma and allergic rhinitis in Pakistani patients. J InvestigAllergolClinImmunol.

[10] Fumagalli M, Pozzoli U, Cagliani R (2009). Parasites represent a major selective force for interleukin genes and shape the genetic predisposition to autoimmune conditions. J Exp Med.

[11] Zhao A, McDermott J, Urban JF (2003). Dependence of IL-4, IL-13, and nematode-induced alterations in murine small intestinal smooth muscle contractility on Stat6 and enteric nerves. Immunol.

[12] Yasuda K, Nakanishi K (2018). Host responses to intestinal nematodes. IntImmunol.

[13] Zhu J (2015). T helper 2 (Th2) cell differentiation, type 2 innate lymphoid cell (ILC2) development and regulation of interleukin-4 (IL-4) and IL-13 production. Cytokine.

[14] King IL, Mohrs M (2009). IL-4-producing CD4+ T cells in reactive lymph nodes during helminth infection are T follicular helper cells. J Exp Med.

[15] Baqai R (1996). Role of cytokines in parasitic disease. J Pak Med Assoc.

[16] Luoni G, Verra F, Arca B (2001). Antimalarial antibody levels and IL4 polymorphism in the Fulani of West Africa. Genes Immun.

[17] Nakashima H, Miyake K, Inoue Y (2002). Association between IL-4 genotype and IL-4 production in the Japanese population. Genes Immun.

[18] Gyan BA, Goka B, Cvetkovic JT (2004). Allelic polymorphisms in the repeat and promoter regions of the interleukin 4 gene and malaria severity in Ghanaian children. Clin Exp Immunol.

[19] Verra F, Luoni G, Calissano C (2004). IL4-589C/T polymorphism and IgE levels in severe malaria. Acta Trop.

[20] Rockman MV, Hahn MW, Soranzo N, Goldstein DB, Wray GA (2003). Positive selection on a human-specific transcription factor binding site regulating IL4 expression. Curr Biol.

[21] Nakayama EE, Hoshino Y, Xin X (2000). Polymorphism in the interleukin-4 promoter affects acquisition of human immunodeficiency virus type 1 syncytium-inducing phenotype. J Virol.

[22] Naslednikova IO, Konenkov VI, Ryazantseva NV (2007). Role of genetically determined production of immunoregulatory cytokines in immunopathogenesis of chronic viral hepatitides. Bull ExpBiol Med.

[23] Basehore MJ, Howard TD, Lange LA (2004). A comprehensive evaluation of IL4 variants in ethnically diverse populations: association of total serum IgE levels and asthma in white subjects. J Allergy Clin Immunol.

[24] Paffen E, Medina P, de Visser MC (2008). The− 589C> T polymorphism in the interleukin-4 gene (IL-4) is associated with a reduced risk of myocardial infarction in young individuals. J ThrombHaemost.

[25] Anthony RM, Rutitzky LI, Urban JF, Stadecker MJ, Gause WC (2007). Protective immune mechanisms in helminth infection. Nat Rev Immunol.

[26] Clough D, Kappeler PM, Walter L (2011). Genetic regulation of parasite infection: empirical evidence of the functional significance of an IL4 gene SNP on nematode infections in wild primates. Front Zool.

[27] Daniel WW (1999). Biostatistics: a foundation for analysis in the Health Sciences.

[28] Sambrook J, Fritschi EF, Maniatis T (2017). Molecular cloning: a laboratory manual.

[29] Interactive Research and Development (2017). Base line survey report of soil transmitted helminths prevalence in Pakistan.

[30] Owaka EE, Njoku OO, Uhuo CA, Odikamnoro OO (2016). Survey of intestinal helminth infection amongst school children in rural communities of Ebonyi state Nigeria. Inter J Sci Res Pub.

[31] Tasawar Z, Kausar S, Lashari MH (2010). Prevalence of Entamoebahistolytica in humans. Pak J Pharm Sci.

[32] Galgamuwa LS, Iddawela D, Dharmaratne SD (2018). Prevalence and intensity of Ascarislumbricoides infections in relation to undernutrition among children in a tea plantation community, Sri Lanka: a cross-sectional study. BMC Pediatr.

[33] Rivero MR, De Angelo C, Nuñez P (2017). Environmental and socio-demographic individual, family and neighborhood factors associated with children intestinal parasitoses at Iguazú, in the subtropical northern border of Argentina. PLoS Negl Trop Dis.

[34] Gelaw A, Anagaw B, Nigussie B (2013). Prevalence of intestinal parasitic infections and risk factors among schoolchildren at the University of Gondar Community School, Northwest Ethiopia: a cross-sectional study. BMC Public Health.

[35] Osei A, Houser R, Bulusu S, Joshi T, Hamer D (2010). Nutritional status of primary schoolchildren in Garh-wali Himalayan villages of India. Food Nutr Bull.

[36] Galgamuwa LS, Iddawela D, Dharmaratne SD, Galgamuwa GL (2017). Nutritional status and correlated socio-economic factors among preschool and school children in plantation communities, Sri Lanka. BMC Public Health.

[37] Ramzan M, Ali I (2008). Body mass status of school children of Dera Ismail Khan, Pakistan. J Ayub Med Coll Abbottabad.

[38] Maia MM, Fausto MA, Vieira EL, Benetton ML, Carneiro M (2009). Intestinal parasitic infection and associated risk factors, among children presenting at outpatient clinics in Manaus, Amazonas state, Brazil. Ann Trop Med Parasitol.

[39] Suntaravitun P, Dokmaikaw A (2018). Prevalence of intestinal parasites and associated risk factors for infection among rural communities of Chachoengsao Province, Thailand. Korean J Parasitol.

[40] Siddiqui MI, Bilqees FM, Iliyas M, Perveen S (2002). Prevalence of parasitic infections in a rural area of Karachi, Pakistan. J Pak Med Assoc.

[41] Salawu SA, Ughele VA (2015). Prevalence of soil-transmitted helminths among school-age children in Ife East Local Government Area, Osun State, Nigeria. FUTA J Res Sci.

[42] Nasr NA, Al-Mekhlafi HM, Ahmed A, Roslan MA, Bulgiba A (2013). Towards an effective control programme of soil-transmitted helminth infections among Orang Asli in rural Malaysia. Part 2: Knowledge, attitude, and practices. Parasit Vectors.

[43] Bhardwaj S, Sambu W, Jamieson L (2017). Setting an ambitious agenda for children: The Sustainable Development Goals. South African child guage.

[44] Faria CP, Zanini GM, Dias GS (2017). Geospatial distribution of intestinal parasitic infections in Rio de Janeiro (Brazil) and its association with social determinants. PLoSNegl Trop Dis.

[45] Kumar S, Singh J, Kumar A (2017). Prevalence and correlation of soil transmitted helminth infection to the degree of anemia and nutritional status among pediatric patients of age group 6–14 years in Kishanganj, Bihar, India. Int J Contemp Pediatrics.

[46] Strunz EC, Addiss DG, Stocks ME, Ogden S, Utzinger J, Freeman MC (2014). Water, sanitation, hygiene, and soil-transmitted helminth infection: a systematic review and meta-analysis. PLoS Med.

[47] Kabesch M, Tzotcheva I, Carr D (2003). A complete screening of the IL4 gene: novel polymorphisms and their association with asthma and IgE in childhood. J Allergy Clin Immunol.

[48] Finkelman FD, Shea-Donohue T, Morris SC (2004). Interleukin-4 and interleukin-13 mediated host protection against intestinal nematode parasites. Immunol Rev.

[49] Qi H, Sun L, Jin YQ (2014). rs2243268 and rs2243274 of Interleukin-4 (IL-4) gene are associated with reduced risk for extrapulmonary and severe tuberculosis in Chinese Han children. Infect Genet Evol.

[50] Baye TM, Kovacic MB, Myers JM (2011). Differences in candidate gene association between European ancestry and African American asthmatic children. PloS One.

[51] Maier LM, Chapman J, Howson JM (2005). No evidence of association or interaction between the IL4RA, IL4, and IL13 genes in type 1 diabetes. Am J Hum Genet.

[52] Naka I, Nishida N, Patarapotikul J (2009). Identification of a haplotype block in the 5q31 cytokine gene cluster associated with the susceptibility to severe malaria. Malar J.

[53] Arnez LE, Venegas EN, Ober C, Thompson EE (2011). Sequence variation in the IL4 gene and resistance to Trypanosomacruzi infection in Bolivians. J Allergy Clin Immunol.

[54] Rong H, He X, Wang L, He Y, Kang L, Jin T (2017). Associations between polymorphisms in the IL-4 gene and renal cell carcinoma in Chinese Han population. Oncotarget.

[55] Lee YJ, Huang CY, Ting WH (2011). Association of an IL-4 gene haplotype with Graves disease in children: experimental study and meta-analysis. Hum Immunol.

[56] Wang J, Chen R, Tang S (2015). Interleukin-4 and interleukin-10 polymorphisms and antituberculosis drug-induced hepatotoxicity in C hinese population. J Clin Pharm Ther.

[57] Jin T, Zhang Y, Sun Y, Wu J, Xiong Z, Yang Z (2019). IL-4 gene polymorphisms and their relation to steroid-induced osteonecrosis of the femoral head in Chinese population. Mol Genet Genomic Med.

[58] Joubert BR, Reif DM, Edwards SW (2011). Evaluation of genetic susceptibility to childhood allergy and asthma in an African American urban population. BMC Med Genet.

[59] Miyake Y, Tanaka K, Arakawa M (2012). Polymorphisms in the IL4 gene, smoking, and rhinoconjunctivitis in Japanese women: The Kyushu Okinawa Maternal and Child Health Study. Hum Immunol.

[60] Bondurant KL, Lundgreen A, Herrick JS, Kadlubar S, Wolff RK, Slattery ML (2013). Interleukin genes and associations with colon and rectal cancer risk and overall survival. Int J Cancer.

[61] Lupianez CB, Canet LM, Carvalho A (2016). Polymorphisms in host immunity-modulating genes and risk of invasive aspergillosis: results from the AspBIOmics consortium. Infect Immun.

[62] Lin Y, He Y (2014). The ontology of genetic susceptibility factors (OGSF) and its application in modeling genetic susceptibility to vaccine adverse events. J Biomed Semantics.

[63] Aschebrook-Kilfoy B, Zheng T, Foss F (2012). Polymorphisms in immune function genes and non-Hodgkin lymphoma survival. J Cancer Surviv.

